# Cardiovascular risk among community members in three communities in the Cape Metropole of the Western Cape

**DOI:** 10.4102/phcfm.v16i1.4246

**Published:** 2024-05-14

**Authors:** Talitha Crowley, Rukshana Francis, Tasneem Ismail, Jeffrey Hoffman, Thabani M. Noncungu, Jennifer A. Chipps

**Affiliations:** 1 School of Nursing, Faculty of Community and Health Sciences, University of the Western Cape, Cape Town, South Africa

**Keywords:** blood pressure, cardiovascular disease, cardiovascular risk, community, risk factors, screening, weight

## Abstract

**Background:**

Cardiovascular diseases pose a risk to population health in South Africa and are responsible for almost one in six deaths (17.3%).

**Aim:**

To determine the cardiovascular risk among community members who attended a community outreach programme.

**Setting:**

Three communities in the Cape Metropole of the Western Cape.

**Methods:**

A health survey was conducted with 783 participants, 18 years and older, conveniently sampled. The survey included questions about cardiovascular risk factors, and biometric measurements of blood pressure (BP), height and weight were conducted.

**Results:**

A total of 777 participants were included in the study. Most participants were female (529, 68.1%), with an average age of 42.3 years (s.d. 14.2). Risk behaviours reported included smoking (216, 27.8%), consuming more than two drinks of alcohol daily (78, 10%), low physical activity (384, 49.4%), being stressed on most days (436, 56.1%) and unhealthy eating habits (253, 32.6%). More than half of the participants (402, 51.7%) had a body mass index (BMI) ≥ 30, 26.0% (202) had a systolic BP of ≥ 140 mm Hg and 22.4% (174) had a diastolic BP of ≥ 90 mm Hg; 16.6% (130) had a cardiovascular disease (CVD) risk of 10–20 and 19.3% (150) had a CVD risk of > 20%.

**Conclusion:**

Nearly a fifth of the participants had a significant probability of developing heart disease or experiencing a stroke over the next 10 years.

**Contribution:**

There is an urgent need for comprehensive health promotion and behaviour change interventions focused on reducing CVD risk factors at the community level.

## Introduction

Cardiovascular diseases (CVDs) pose a risk to population health, both globally and in South Africa.^1^ Cardiovascular diseases, commonly referred to as heart disease or stroke, are the number one cause of death around the world with a reported one in three deaths globally, yet most premature heart disease and stroke are preventable.^1^ Over three-quarters of heart diseases and stroke-related deaths occur in low- and middle-income countries such as South Africa.^2^ Although previously perceived as a disease that affects the elderly, more than 50% of mortalities occur before the age of 65 years.^3^

In the second National Burden of Disease study based on data from 1997 to 2012, cerebrovascular disease was the leading cause of death in South Africa after HIV and AIDS.^4^ In 2016, the Heart and Stroke Foundation of South Africa reported that CVD was responsible for almost 1 in 6 deaths, amounting to 17.3%.^5^ The data also indicated a daily average of 215 deaths attributed to heart disease or stroke.^5^ Notably, the report revealed that every hour, 5 people have heart attacks and 10 people have strokes, of which 10 people die because of these events.^5^ The most recent data on the causes of death in South Africa for the year 2019 unveiled that diseases of the circulatory system emerged as the predominant category, accounting for 17.5% of all reported deaths.^6^ Of the top 10 underlying causes of mortality, 3 were cardiovascular-related diseases: cerebrovascular diseases (ranked third), hypertensive disease (ranked fifth) and ischaemic heart diseases (ranked seventh).^6^ Among females, cerebrovascular disease was ranked the second leading cause of death followed by hypertensive disease.^6^

The high prevalence of CVD is supported in a recent systematic review, which reported the overall South African national prevalence of coronary heart disease as 1.29 (95% CI = 0.83; 1.75) and 4.29 (95% CI = 3.13; 5.45) for stroke.^7^ A study conducted among older adults in South Africa using the Framingham risk scores (FRS) found a 10-year CVD risk of 17%, which was significantly higher in men compared with women (23.2% vs. 14.3%, *p* < 0.001).^8^ A pilot study on CVD risk conducted among 489 teachers in Cape Town found that 18.7% were at high risk of a heart attack or stroke within 10 years.^9^

South Africa has a heavy burden of hypertension with an estimated prevalence between 27% and 58%.^10^ Differences in prevalence have been reported across various settings. In a rural community in the Eastern Cape, 71% of participants were hypertensive.^11^ Peer et al. found an age-standardised prevalence of 38.9% in an urban population in Cape Town.^12^ The burden of hypertension will increase substantially without effective intervention strategies across the treatment cascade. This includes screening, prevention through the identification of modifiable risk factors, early diagnosis, treatment and follow-up.^12^

The early detection and management of CVD risk may prevent mortality.^5^ An overview of systematic reviews on the effect of using cardiovascular risk scoring in routine risk assessment in primary prevention of CVD found a tendency towards reduction of blood pressure (BP), total cholesterol and smoking levels, especially in high-risk patients.^13^ Jardim et al. aver that CVD is not being optimally managed in some areas of South Africa with significant disparities in the control of CVD risk factors.^3^ High mortality rates in CVD may stem from inadequate control of risk factors. By enhancing the management of these factors and incorporating community education on lifestyle modification, mortality rates could be effectively reduced.^3^

The risk factors for CVD include hypertension, overweight and obesity, high cholesterol, diabetes, tobacco use, physical inactivity, unhealthy diet and salt intake.^14^ As many people with hypertension and diabetes are unaware of the condition, it often remains undiagnosed, untreated and uncontrolled.^15,16,17^ Berry et al. found that among South Africans aged 15 years and older who had hypertension, 48.7% remained unscreened and undiagnosed, 23.1% underwent screening but remained undiagnosed, 5.8% were diagnosed but not receiving treatment, and 13.5% were receiving treatment but had uncontrolled hypertension.^16^ About 65.1% of women and almost 31.2% of men are overweight in South Africa and 40.1% of women are obese compared with 11.6% of men.^5^ Those who smoke and those exposed to second-hand smoke have an increased risk of developing CVD.^5^ In addition, physical inactivity contributes much to the increased risk of CVD, especially in our modern world of sedentary lifestyles.^5^ In a Cape Town study among teachers, CVD risk factors included hypertension (48.5%), hypercholesterolaemia (20.5%), smoking (18.0%), diabetes (10.1%) and being overweight or obese (84.7%).^9^

The high prevalence of CVD risk factors in communities and poor access to healthcare may lead to delayed diagnosis and management of CVD risk.^18^ Initiatives such as population-wide screening for hypertension and diabetes, engagement of community resources and governance structures as well as geographic decentralisation of care services have been proposed, but the evidence of effectiveness is still lacking.^19,20^ In certain communities, community health workers have been successfully trained to conduct cardiovascular risk assessments.^21^ However, a qualitative study on the knowledge and perceptions of cardiovascular risk in a low-income peri-urban community in the Western Cape found that community members had a poor understanding of risk and that this understanding is key to risk assessments in the community.^22^

This study was conducted as part of a community outreach project focused on CVD risk identification and prevention. The aim of this study was to determine the cardiovascular risk among community members who attended an outreach programme in three communities in the Cape Metropole of the Western Cape in order to conduct targeted referrals and behaviour change counselling.

## Research methods and design

### Study design

The study design was cross-sectional. A survey questionnaire and anthropometric and biometric measures were used.

### Setting

Three outreach communities affiliated with the Faculty of Community and Health Sciences (CHS) of the University of the Western Cape (UWC) were included in the study. Community A is a village of approximately 2300 people situated 15 km north of Durbanville. Community B is a township located 10 km northeast of Durbanville. It has a population of 12 369 people. Community C has a population of approximately 112 507 people according to the 2011 Census.

The data collection site in Community C is a bustling area and is situated adjacent to a taxi rank with people from various other communities accessing it. The three main languages in the communities are Afrikaans, English and Xhosa.

### Population and sampling

Willing participants were conveniently sampled when attending designated wellness stations set up during the outreach in the three communities. Participants were recruited through the distribution of flyers in the community. The inclusion criteria were all adults (≥ 18 years), irrespective of preexisting conditions who were willing to participate. Participants under the age of 18 years were excluded.

### Sample size

For an unknown population with an unknown prevalence of CVD risk (50%) and a confidence limit of ± 5%, a sample size of approximately 384 participants was calculated when random sampling was assumed. For a risk of 18% as reported in a previous study conducted in Cape Town,^9^ a sample size of 227 was needed.

### Instrument and measures

We developed a brief survey to collect data on cardiovascular risk factors based on the literature and practice guidelines, the Practical Approach to Care Kit Adult,^23^ used by primary care nurses in the Western Cape. Questions included sex, age, smoking, alcohol consumption, stress, exercise, eating habits and personal and/or family history of hypertension, diabetes or CVD. Definitions for key terms were provided based on the guidelines^23^; for example, ‘one drink is 1 tot of spirits or 1 small glass (125 mL) of wine or 1 can/bottle (330 mL) of beer’ or ‘moderate exercise is brisk walking, jogging, cycling, swimming, playing sports or any exercise that increases breathing and heart rate continuously for at least 30 minutes’:

Healthy eating habits include eating a variety of foods in moderation. Daily fruit, vegetables, nuts and legumes. Choosing whole grain bread/rice or potatoes rather than white bread/rice. Replacing brick margarine/butter with vegetable oil or soft tub margarine. Removing skin and fat from meat. Reducing salty processed foods like gravies, stock cubes, and packet soup. Avoid adding salt to food. Avoiding or using less sugar.

Response options were binary (yes or no). No data on current treatment or adherence were collected. (Refer to Online Appendix 1 for the survey tool.) In addition, we measured the following biometric measures: BP and weight and height, which were used to calculate body mass index (BMI). Body mass index was calculated using the formula: BMI = weight (kg) ÷ height (m) ÷ height (m). Blood pressure screening was performed in a sitting position with standardised BP machines, and height and weight measurement was carried out with standard height measurement tapes and scales routinely used during outreaches. Scales were calibrated using a 5 kg weight. As a result of the pragmatic nature of the study in the context of outreach, all measurements were only taken once. Abnormal measures were performed twice.

We used the Framingham Heart Study general CVD risk prediction calculator (FRS) using the BMI to calculate CVD risk.^24^ The FRS makes provision for substituting lipids for BMI. The calculator is based on an online risk calculator (in Excel) that includes values for sex, age, systolic BP, treatment for hypertension, current smoker, diabetes and BMI to establish the 10-year risk of the participant to develop CVD.^24^ If CVD risk is < 10%, there is a less than 1 in 10 chance that they may have a heart attack or stroke over the next 10 years; 10% – 20%, there is a 1–2 in 10 chance that they may have a heart attack or stroke over the next 10 years; and > 20%, there is a more than 2 in 10 chance that they may have a heart attack or stroke over the next 10 years.^23^

### Data collection procedures

Data were collected from 11 April 2023 to 14 April 2023. Fourth-year Bachelor of Nursing students supervised by clinical facilitators (qualified professional nurses registered with the South African Nursing Council) collected data from participants. The students were trained by the clinical facilitators and the research team to complete the survey and conduct the measurements.

The completion of the questionnaire and measurements took approximately 10 min. If the calculated CVD risk was ≥ 10%, the participant was referred to the nearest primary healthcare (PHC) clinic for CVD risk routine care. If the CVD risk was < 10%, the participant was advised to go for a reassessment of their CVD risk after 5 years and provided advice about their general health. Participants with high BP, for example, a systolic BP of ≥ 140 mm Hg or a diastolic BP of ≥ 90 mm Hg, were referred. All the participants received a handheld card with their measures for use at the next clinic visit. Results were explained to the participants by the student nurse and/or clinical facilitator. Participants were offered on-site brief behaviour change counselling if there were any CVD risk factors present and if they had a high CVD risk. A total of 248 participants were referred to their nearest PHC clinic and 202 also received on-site counselling.

### Validity and reliability

Validity and reliability were ensured by calibrating the scales for the physical measurements, training students on the data collection measures and close supervision of all physical measurements. Abnormal measures were checked twice. We used existing measures to calculate BMI and CVD risk assessment as per the guidelines. A BMI between 25.0 and 29.9 was considered overweight and > 30.0, obese.^23^

### Data analysis

Data collected from hard copy questionnaires were checked for accuracy by the on-site clinical facilitators or a member of the research team and captured by a data capturer in the Statistical Programme for the Social Sciences (SPSS, version 28). Data were analysed by gender and six records with no gender information were excluded. Demographic and risk category data were described using descriptive statistics, and chi-square (*X*^2^) and independent samples t-tests were used to explore differences between the genders. During the data cleaning process, the risk calculation was verified by comparing the raw data against the instructions provided for the Framingham Heart Study general CVD risk prediction calculator, commonly known as the FRS.^23^

### Ethical considerations

An application for full ethical approval was made to the University of the Western Cape BMREC and ethics consent was received on 13 October 2022. The ethics approval number is BM22/8/14. The Western Cape Department of Health also approved the research study. The approval number is WC_202302_041. Meetings were held with the clinical managers of the clinics in the three communities to arrange for the referral of participants. Written informed consent was obtained from participants in Afrikaans, English or Xhosa. Participants who did not consent to participate in the research study were still eligible for screening services and referral, but their information was not included in the study. No participant-identifying details, for example, names, were collected on questionnaires and only participant numbers were used.

## Results

### Demographic data

A total of 783 participants participated, but only 777 were included in the study, with 6 participants excluded based on lack of gender information. Most participants were female (529, 68.1%), with an average age of 42.3 years (standard deviation [s.d.] 14.2), ranging from 18 to 83 years. Nearly a third of the participants (231, 28.7%) were 50 years and older (Table 1).

**TABLE 1:** Demographic data.

Characteristic	Male (*n* = 248)				Female (*n* = 529)				All (*N* = 777)				Test	*p*
	*n*	%	Mean	s.d.	*n*	%	Mean	s.d.	*n*	%	Mean	s.d.		
Community A	30	26.3	-	-	84	73.7	-	-	114	14.7	-	-	*X*^2^ = 4.4	0.110
Community B	34	27.0	-	-	92	73.0	-	-	126	16.2	-	-	-	-
Community C	184	34.3	-	-	353	65.7	-	-	537	69.1	-	-	-	-
Age (years)	-	-	42.6	14.3	-	-	42.0	14.2	-	-	42.3	14.2	*T* = 0.3	0.732
≥ 50 years	84	33.9	-	-	147	27.8	-	-	231	29.7	-	-	*X*^2^ = 3.0	0.084

### Cardiovascular risk factors

This study examined several key cardiovascular risk factors (Table 2). About half of the participants (384, 49.4%) reported not participating in moderate exercise, and nearly a third (252, 32.6%) reported not eating healthily. In terms of substance use, nearly a third of the participants reported being active smokers (216, 27.8%) and drinking more than two alcoholic drinks a day (78, 10.0%). Male participants had significantly higher levels of smoking and drinking than female participants. Nearly half of the male participants reported drinking more than two alcoholic drinks a day (51, 20.6%) (*X*^2^ = 68.5, *p* < 0.001) compared with only 27 (5.1%) of female participants. More than a third (97, 39.1%) of males reported being current smokers compared with 119 (22.5%) female participants (*X*^2^ = 23.2, *p* < 0.001). More than half of the participants (436, 56.1%) reported feelings of stress on most days, with more female respondents reporting stress compared with males (314, 59.4% vs. 122, 49.2%, respectively, *X*^2^ = 7.1, *p* = 0.008).

**TABLE 2:** Cardiovascular risk factors.

Cardiovascular risk factor	Male (*n* = 248)				Female (*n* = 529)				All (*N* = 777)				Test	*p*
	*n*	%	Mean	s.d.	*n*	%	Mean	s.d.	*n*	%	Mean	s.d.		
No moderate exercise at least 5 days a week	111	44.8	-	-	273	51.6	-	-	384	49.4	-	-	*X*^2^ = 3.1	0.075
Eating habits or diet not considered healthy	80	32.3	-	-	173	32.7	-	-	253	32.6	-	-	*X*^2^= 0.02	0.902
Current smoker	97	39.1	-	-	119	22.5	-	-	216	27.8	-	-	*X*^2^ = 23.2	< 0.001
Alcohol > 2 drinks per day	51	20.6	-	-	27	5.1	-	-	78	10.0	-	-	*X*^2^= 68.5	< 0.001
Feeling stressed on most of the days	122	49.2	-	-	314	59.4	-	-	436	56.1	-	-	*X*^2^ = 7.1	0.008
Systolic BP (mm Hg)	-	-	132.5	17.0	130.5	-	130.5	16.7	-	-	131.4	16.8	*T* = 1.5	0.125
Diastolic BP (mm Hg)	-	-	80.9	12.9	80.4		80.4	11.8			80.6	12.2	*T* = 5.6	0.552
Systolic (≥ 140 mm Hg)	78	31.5	-	-	124	23.4	-	-	202	26.0	-		*X*^2^ = 5.6	0.018
Diastolic (≥ 90 mm Hg)	61	24.6	-	-	113	21.4	-	-	174	22.4	-		*X*^2^ = 1.0	0.313
Hypertension treatment	49	19.8	-	-	133	25.1	-	-	182	23.4	-		*X*^2^ = 2.8	0.099
Current diabetes	13	5.2	-	-	44	8.3	-	-	57	7.0	-		*X*^2^ = 2.3	0.125
Family history diabetes	67	27.0	-	-	166	31.4	-	-	233	30.0	-	-	*X*^2^ = 1.5	0.216
Cardiovascular disease or heart attack or stroke	21	8.5	-	-	38	7.2	-	-	59	7.6	-	-	*X*^2^ = 0.4	0.523
Family history of heart disease or stroke	63	25.4	-	-	141	26.7	-	-	204	26.3	-	-	*X*^2^ = 0.2	0.712
BMI ≥ 30	56	22.6	-	-	346	65.4	-	-	402	51.7	-	-	*X*^2^ = 124.0	< 0.001
BMI	-	-	26.60	5.9	-	-	33.40	7.9	-	-	31.18	7.9	*T* = 12.1	< 0.001

BMI, body mass index; BP, blood pressure.

The overall average BMI was 31.18 kg/m^2^ (s.d. 7.9), which is in the obese range. Female participants had significantly higher BMIs, with an average of 33.4 kg/m^2^ (s.d. 7.9) compared with male participants with an average of 26.6 kg/m^2^ (s.d. 5.9). Furthermore, 346 (65.4%) females were in the obese weight range compared with 56 (22.6%) males (*X*^2^ = 65.7, *p* < 0.001). Male participants had significantly higher average systolic BPs, with 71 (31.5%) compared with 124 (23.4%) found to have BP ≥ 140 mm Hg, *X*^2^ = 5.6, = 0.018. Only 57 (7.3%) of the participants reported having a diagnosis of diabetes, although 233 (30.0%) reported having a family history of diabetes.

Nearly a quarter of participants reported receiving treatment for hypertension (182, 23.4%) and just over half (101, 55.5%) of those had a controlled systolic BP. Of the participants with a systolic BP ≥ 140 mm Hg (202), only 81 (40.1%) were on treatment for hypertension. This means there is potentially a high number of persons with untreated hypertension in the communities.

### Cardiovascular risk

The overall cardiovascular risk was high with 19.3% (150) of the participants having a risk of more than 20.0%. This means a 2 in 10 chance that they may have a heart attack or stroke over the next 10 years (Table 3). This risk was significantly higher in males (90, 36.3%) compared with female participants (60, 11.3%), with *X*^2^ = 64 and *p* < 0.001. Similar patterns were seen with participants with a cardiovascular risk between 10.0% and 20.0% (51, 20.6% vs. 79, 14.9%; *U* = 82.3, *p* < 0.001).

**TABLE 3:** Cardiovascular disease risk.

Cardiovascular risk	Male (*n* = 248)		Female (*n* = 529)		All (*N* = 777)		Test	*p*
	*n*	%	*n*	%	*n*	%		
> 20%	90	36.3	60	11.3	150	19.3	*X*^2^ = 82.3	< 0.001
10% – 20%	51	20.6	79	14.9	130	16.6	-	-
< 10%	107	43.1	390	73.7	497	63.5	-	-

## Discussion

Our study found that more than a third (35.9%) of community members (mean age 42.3 years) who attended an outreach programme in three communities in the Cape Metropole of the Western Cape had intermediate (16.6%) or high (19.3%) CVD risk. This is slightly lower than another South African study among older adults (≥ 50 years) in KwaZulu-Natal, where 33.0% of participants had low CVD risk (FRS < 10%), 39.0% intermediate risk (FRS 10.0% – 19.0%) and 28.0% high risk (FRS ≥ 20.0%).^8^ The risk reported in our study is similar to the CVD risk reported among teachers (mean age 46.3 years) in Cape Town where 18.7% of participants had a high (> 20.0%) 10-year CVD risk calculated using the World Health Organization risk predictor charts.^9^ In the same study, the risk was lower (13.9%) when the Framingham risk calculator using the BMI was used.^9^ The risk was significantly higher for males compared with females, which is similar to the findings of the KwaZulu-Natal study.^8^

The Framingham risk calculator includes several factors to estimate an individual’s CVD risk. These factors are age, gender, BMI or cholesterol levels, BP, treatment for hypertension, smoking status and the presence of diabetes.^24^ Cardiovascular diseases risk factors in this study included high levels of stress and low levels of participation in moderate exercise. Of note is that more than half of the participants were obese (BMI ≥ 30); 65.4% of females and 22.6% of males and females had significantly higher BMIs than males. These findings were higher than the South African prevalence for obesity (50% for women and 23% for males) reported in the World Obesity Atlas 2022.^25^ However, it was lower than the findings reported among Cape Town teachers, which found that 84.7% of participants were obese.^9^ Andersson et al. observe that a high prevalence of CVD risk factors, such as obesity, physical inactivity and poor diet, has been observed among young individuals (age 18–50 years) living in developed countries.^26^ This has led to an increase in CVD risk among the younger population, which is also attributable to the higher prevalence of substance use, for example, tobacco smoking and alcohol use.^26^ When compared with the global burden of CVD, people in Africa are typically younger, predominantly female and mostly from disadvantaged communities.^27^ Our study found markedly higher levels of stress among females compared with males, which could be a contributing factor to CVD. Even though CVD is the leading cause of death among women globally, it remains understudied.^28^ The Lancet Commission recommended prioritising funding for CVD health programmes in women from socioeconomic deprived regions and educating healthcare providers and young women regarding the prevention and early detection of CVD among women.^28^ Despite this, the CVD risk was significantly higher among males compared with females. When comparing the risk factors, significantly more males compared with females had a systolic BP of ≥ 140 mm Hg, smoked and consumed alcohol.

In our study, 23.4% of participants were on treatment for hypertension and 26.0% had a systolic BP of > 140 mm Hg. According to the South African National Health and Nutrition Examination Survey in 2012 and the South African Demographic Health Survey (DHS) in 2016, hypertension prevalence was 38.4% and 48.2%, respectively.^29^ Our study found a large percentage of persons with potentially untreated hypertension. Similarly, Berry et al. found that 48.7% of people in South Africa with hypertension were undiagnosed and 13.5% were receiving treatment but had uncontrolled hypertension.^16^

In the DHS 2016, the observed diabetes prevalence was 22.0%,^30^ which is higher compared with our study (7.6%). However, we only relied on reported diagnoses and did not test for diabetes in this study. A Cape Town study among teachers reported a diabetes prevalence of 10.1%.^9^

The Prospective Urban Rural Epidemiology (PURE) study across 21 countries identified that 70% of CVD risk factors are modifiable.^15^ Behavioural risk factors include low levels of education and ambient air pollution. In low- and middle-income countries, household air pollution, poor diet and low education strongly affect CVD and mortality.^15^ Current evidence suggests that hypertension is a multifactorial condition, which is influenced by risk factors such as genetics (non-modifiable factors), sociodemographic and behavioural factors, which can potentially be modified. Furthermore, socioeconomic status is reported to be associated with high BP, awareness of hypertension, health behaviour and access to preventative interventions and measures.^11^

Various socioeconomic factors impact the life course of CVD, which is often beyond the individual’s control.^27^ In order to ensure the global goal of a 25% reduction in premature CVD mortality, better evidence-based policies such as tobacco control are needed as well as CVD prevention and management in PHC and community settings. These efforts should be coupled with better health information systems to monitor progress.^31^

The Western Cape Department of Health has recently issued a circular on the recording and reporting of hypertension and diabetes screening (H77/2023), acknowledging the importance of early detection of disease and reducing the hypertension treatment gap (Western Cape Department of Health, Circular H77/2023, personal communication dated 14 June 2023). This is a necessary step towards improving the monitoring of CVD risk in communities, although risk screening should be performed beyond PHC facilities and services.

### Recommendations

Our study showed that it is possible to do low-cost CVD screening on a community level using a brief screening tool. Additional measures such as waist circumference, rapid glucose tests or HbA1c could be incorporated in future outreaches. Furthermore, detailed measurements of diet and exercise can be used to measure these risk factors more accurately. Future studies should explore how behaviour change interventions can be tailored for specific communities.

### Strengths and limitations

This study has a large sample size and was conducted across three communities measuring BP, BMI and CVD risk at the time of data collection. A limitation is the utilisation of convenience sampling, which may have introduced selection bias and resulted in a lack of diversity in terms of age and sex. In addition, anthropometric and biometric measurements were only taken once, except in the case of abnormal readings, which could have affected the reliability of the measures.

## Conclusion

We found high frequencies of cardiovascular risk factors among community members in this study; 19.3% of the participants have a significant probability of developing heart disease or experiencing a stroke over the next 10 years. The risk is significantly higher among males. There is indeed a need for comprehensive health promotion and behaviour change interventions focused on reducing CVD risk factors on a community level.
